# An application of deep learning model *InceptionTime* to predict nausea, vomiting, diarrhoea, and constipation using the gastro-intestinal pacemaker activity drug database (GIPADD)

**DOI:** 10.1038/s41598-025-95961-4

**Published:** 2025-04-16

**Authors:** Hephaes Chuen Chau, Julia Yuen Hang Liu, John Anthony Rudd

**Affiliations:** 1 Gut Rhythm R&D (Hong Kong) Limited, Hong Kong, SAR People’s Republic of China; 2 School of Biomedical Sciences, Faculty of Medicine, The Chinese University of Hong Kong, Lo Kwee-Seong Integrated Biomedical Sciences Building, Shatin, New Territories, Hong Kong, SAR People’s Republic of China

**Keywords:** Deep learning, Artificial intelligence, Gastrointestinal tract, Slow wave activity, Pacemaker activity, Drug discovery, Adverse drug reactions, Vomiting, Nausea, Diarrhoea, Constipation, Drug safety, Drug screening, Data processing, Databases, Machine learning

## Abstract

The accurate preclinical prediction of adverse drug reactions (ADRs), such as nausea and vomiting, remains a challenge. The Gastro-Intestinal Pacemaker Activity Drug Database (GIPADD) (http://www.gutrhythm.com/public_database) is a new source of electrophysiological big data for drug research. Over the past 2 years, the database has doubled in size, and now contains the electrophysiological profiles of 172 drugs across 11,943 datasets. This study used a state-of-the-art deep-learning model with time-series classification to explore the feasibility of using raw electrophysiological recordings from tissues to predict ADRs. The GIPADD contains the recordings of the electrical activity of various gastrointestinal tissues (stomach, duodenum, ileum, and colon) exposed to a drug at three or more different concentrations, representing the effects of the drug on gastrointestinal pacemaker activity. Each drug in the database is associated with at least 60 recordings. The datasets are divided in a ratio of 8:2 for training and validation. A modified *InceptionTime* classifier (ICT) was used to predict whether a drug induces ADRs, using data from the SIDER database as the target. Concentrations and tissues were added as covariates and added to the input of the model during forward propagation. We also established a negative control with shuffled target labels, and external validation was conducted using time-shifted recording predictions. The best model for predicting nausea, vomiting, diarrhoea, and constipation achieved by-drug accuracies of 0.87, 0.89, 0.85, and 0.91, respectively; by-drug precision (*class 1*) of 0.88, 0.90, 0.99, and 0.89, respectively; and area under the receiver operating characteristic curve (AUROC) values of 0.84, 0.87, 0.94, and 0.96, respectively. The best model was an ensemble of five independent ICT classifiers trained on the same dataset. Models trained using shuffled labels (negative controls) exhibited significantly lower accuracy, precision, and AUROC values than models trained using correctly labelled datasets, indicating that ICT classifiers successfully identified latent features in the raw recordings associated with ADRs. The combined benefits of the GIPADD and deep learning may accelerate drug safety testing and drug development by enabling the reliable analysis of electrophysiological drug profiles during the preclinical stage.

**Supplementary Information:**

The online version contains supplementary material available at 10.1038/s41598-025-95961-4.

## Introduction

New drug therapies desired by many patients must first undergo clinical trials before approval. However, these trials often have high dropout rates, mainly due to unforeseen adverse drug reactions (ADRs) that outweigh the benefits of treatment^1^. Nausea and vomiting are among the most common ADRs that impede drug discovery and development in late stages^2,3^. Thus, these ADRs may limit a drug’s adoption even after it enters the market. For decades, nausea and vomiting (28.9%) and small bowel obstruction (19.9%) have been the most common causes of unplanned hospital admissions after chemotherapy^4^. Furthermore, new weight loss and diabetic drugs, such as GLP1 agonists, have high discontinuation rates because of nausea (64.4%) and vomiting (45.4%), as reported by patients, and other gastrointestinal (GI) side effects (36.8%), as reported by physicians^5^. Predicting nausea and vomiting in the preclinical phase is challenging. Currently, this is mainly addressed through animal testing using models capable of vomiting (e.g. dogs or ferrets). However, the use of these animal models for testing vomiting properties before clinical trials is not a universal practice, and while it may detect vomiting, accurate assessment of nausea is not optimal or reliable^6,7^.

Significant efforts have been made to identify vomiting-related biomarkers in genetic or proteomic studies; however, representative biomarkers have not yet been identified. Our previous studies have indicated that drug-induced alterations in GI pacemaker activities (GIPAs), also known as slow wave activities, are potential predictive ‘electrical’ biomarkers of vomiting^8,9^. GIPAs are a form of rhythmic, propagating, electrical activity generated by a network of interstitial cells of Cajal (ICCs) in the GI tract to regulate GI motility^10^. Drugs can modify GIPAs either directly or indirectly by affecting ICCs, neurons, immune cells, and other cell types in the GI tract^11^. These signals can travel through the brain-gut axis, potentially leading to adverse drug reactions such as vomiting and nausea^12^.

We used a microelectrode array platform to efficiently record and screen the effects of drugs on GIPAs^9,13–17^. The data from these studies were compiled in a novel drug database named the Gastro-Intestinal Pacemaker Activity Drug Database (GIPADD), which is based on an animal model capable of vomiting, specifically the Asian musk shrew (*Suncus murinus*). The GIPADD contains data on pure drug-induced changes in the electrophysiology recordings of ex-vivo gut tissues from this animal model, representing the gut’s pacemaker activities. The data consist of recordings from various gut tissues, including the stomach, duodenum, ileum, and colon, before and after the administration of a drug at three selected concentrations. These selected concentrations are close to the drug’s half-maximal excitatory concentration and half-maximal inhibitory concentration, reflecting expected biological responses. To control for biological variance, each drug was associated with at least 60 datasets, and the recordings were obtained in a single laboratory using standardised methodology. The GIPADD is expected to become a sustainable and publicly accessible online database. As of 10 November 2023, this database included 172 drugs, resulting in 11,943 datasets that were used in this study. By the completion of this manuscript on 8 March 2024, the GIPADD had 217 drugs and 14,675 datasets. These analysed datasets are currently available at https://www.gutrhythm.com/public_database.

We previously used 24 manually extracted features to train non-neural-network machine-learning (ML) models for predicting ADRs, using a database from October 2021 that contained information on 89 drugs and 4867 datasets^18^. However, we found that the performance of these simple ML models declined significantly when applied to large testing sets. In this study, we shifted our focus to deep-learning models, specifically a fully convolutional network (FCN)^19^ and the *InceptionTime* (ICT) classifier, which is the state-of-the-art in time-series classification^20^. These models are designed to identify latent features in 60-channel filtered time-series recordings. Differences between our previous ML approach and the current deep-learning approach are summarised in Table 1.

**Table 1:** Summary table comparing the old machine learning approach published in (Liu et al., 2023) and the current deep learning approach used in this study.

	Liu et al., 2023	This study
Size of GIPADD dataset	89 drugs; 4867 datasets	172 drugs; 11,943 datasets
Cutoff time	Oct 2021	Nov 2023
Model used	Machine learning classifier: Naïve Bayes, discriminant analysis, classification tree, knearest neighbors, support vector machine and an ensemble model	Deep learning *tensorflow* classifiers: simple convolutional neural network (CNN), a fully convolutional network (FCN) and an *InceptionTime* classifier (ICT), and an ensemble model using ICT models only
External validation	Not performed	A forward and a backward time-shifted interference dataset
Model accuracy	< 70% (Internal)	< 90% (time-shifted validation)
Features used in training	24 manually extracted	CNN: >8.3k FCN: >320k ICT: >500k

Apart from vomiting, whether other ADRs, such as nausea, diarrhoea, and constipation, are associated with GIPA remains unclear. To explore this, we hypothesised that our trained models could identify latent features associated with specific ADRs. We conducted an experiment where the model was trained under two settings: one in which the training dataset was correctly labelled and the other in which the labels were shuffled. We compared the performance of the model between these two scenarios to establish an evidence-based correlation between GIPAs and the targeted ADRs. In addition, we introduced a new method to test the performance of time-series classification models by creating time-shifted datasets to evaluate the generalisation ability of the trained models.

## Materials and methods

### Pre-processing

**Fig. 1:**
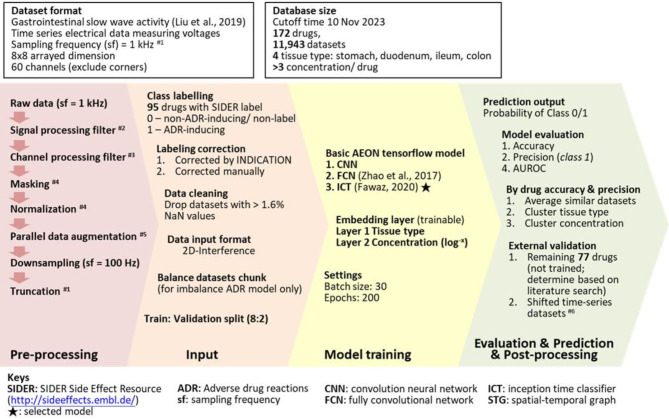
A flowchart depicting raw data preprocessing, input dataset preparation, model training, model prediction, and model evaluation.

**Fig. 2:**
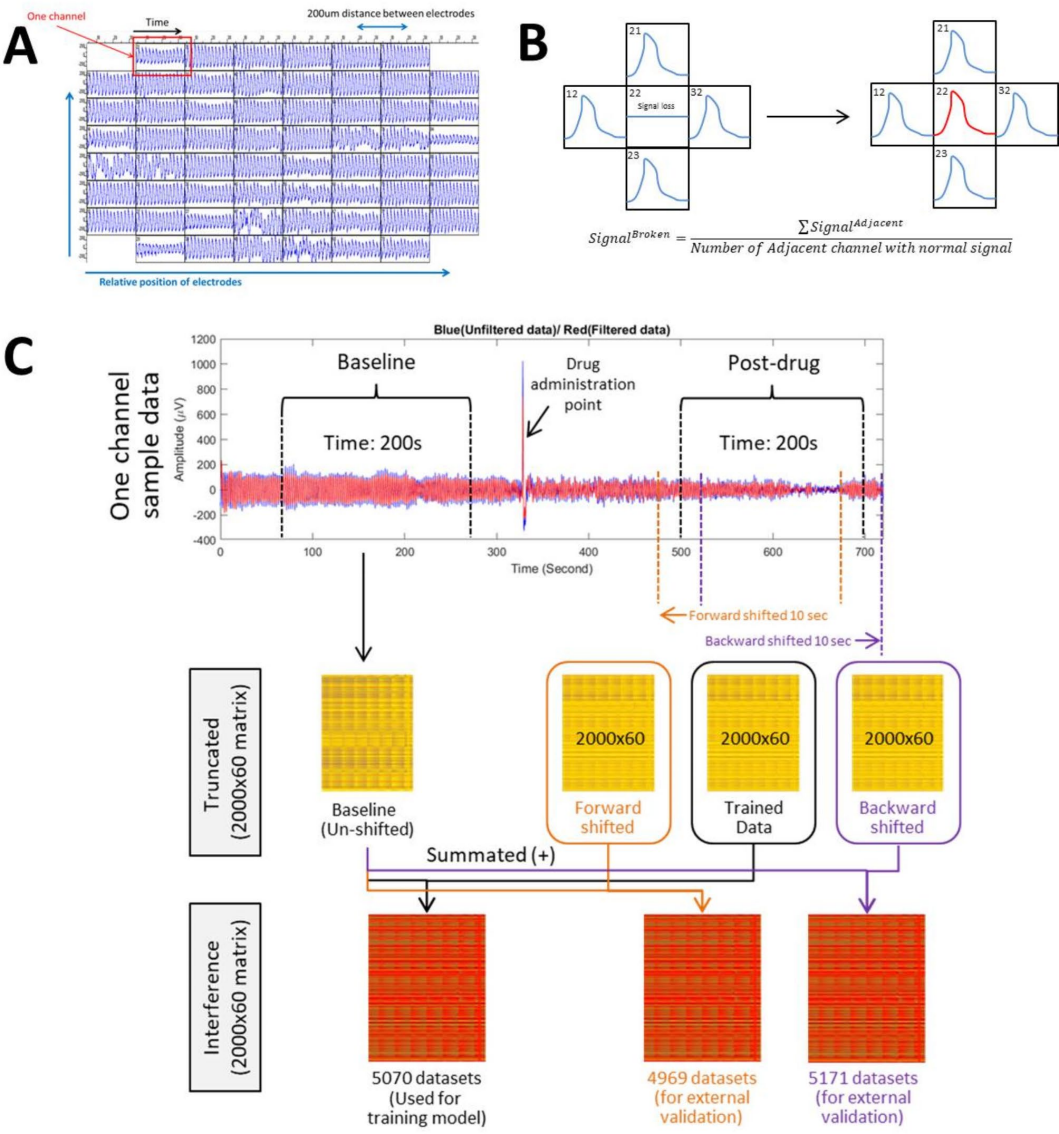
Visual representations of (**A**) the 60-channel time-series electrical voltage data recording gastrointestinal pacemaker activity on an 8 × 8 array platform, (**B**) parallel data augmentation for data cleaning, and (**C**) the creation of the summated ‘interference’ input matrix for model training and time-shifted matrices for external validation. When creating time-shifted matrices for external validation, we shifted only the post-drug signals (200 s), not the baseline signals (200 s). The post-drug signal was trimmed with a 200 s timeframe from the 7-min post-drug recordings. By shifting, we mean to trim the signal using a different timeframe. Forward shifting means the starting time point of the shifted post-drug signals is 10 s earlier than the original post-drug signals used for training models. Backward shifting means the starting time point of the shifted post-drug signals is 10 s later than the original post-drug signals used for training models. Consequently, the time-shifted ‘interference’ datasets will appear completely different from the original ‘interference’ data used for training. Given this distinctiveness, we applied these datasets for external validation.

For this study, only datasets derived from the Asian musk shrew (*S. murinus*), a model capable of vomiting, were extracted from the GIPADD. Each dataset included the covariates t, d, and c, representing the tested tissue, the administered drug, and the drug’s concentration, respectively. The covariates t and c were encoded as integers. Raw data were collected over a duration of 720 s at a sampling frequency (sf) of 1 kHz, with the drug being administered at approximately 300 s (Fig. 1). Subsequently, the raw data underwent signal processing using a MATLAB filter (2*023a; Mathworks*, provided in *SignalProcessingFilter.m*). The processed data were then subjected to a fast Fourier transform (FFT) to identify the dominant frequency (DF) with a bin size of 2048. Channels were then filtered based on the DF thresholds of $\:\ge\:$11 cycles per minute (cpm) for the intestine and $\:\ge\:$4 cpm for the stomach. Any signals exceeding 1500 µV or falling below −1500 µV were masked, considering that the normal signal range is ±25 to 1200 µV and the noise range is ±10 µV. The channel with the highest signal stability during baseline (0–300 s) was determined based on the highest value determined using the following formula: $\:\frac{\sum\:_{DF-1}^{DF+1}Power\:in\:FFT}{\sum\:_{DF=2}^{DF=40}Power\:in\:FFT}$. We calculated the average peak and trough amplitude based on the three most stable channels during the baseline period (0–300 s). The processed data were then normalised to 110% of the average peak and trough amplitude using the following formula:

$\:Normalized\:data=\:\frac{(Filtered\:data-Average\:trough\:amplitude\times\:1.1)}{(Average\:peak\:amplitude\:\times\:1.1-Average\:trough\:amplitude\:\times\:1.1)}$, to ensure that peaks and troughs that are 10% larger than the average were also taken into account without significantly distorting the normalized data. Data augmentation was performed in parallel to augment signals in channels affected by signal loss or filtering, using average signals from adjacent channels (Fig. 2A,B). The data were further downsampled by a factor of 100 and truncated into baseline (50–250 s) and post-drug (500–700 s) matrices, each with dimensions of 2000 × 60 (Fig. 2C).

### Data cleaning and input

The baseline and post-drug recordings were paired to create matched datasets. Those with > 1.6% NaN values in either the baseline or post-drug matrix were excluded. This data cleaning step resulted in approximately 1000 datasets being excluded out of approximately 13,000 matched datasets, leaving 11,943 high-quality datasets. Each matched dataset matrix was then transformed into a summated ‘interference’ matrix by adding the baseline and post-drug matrices together, resulting in a 2000 × 60 dimension $\:\left[\right({2000\times\:60)}_{baseline}+{\left(2000\times\:60\right)}_{postdrug}={\left(2000\times\:60\right)}_{interference}]$ matrix for training all models in this study (Fig. 2C). This summation process ensured the normalisation of all data to the baseline recording, thereby minimising biological variance between animal models.

### Class labelling and labelling correction

ADR class labels were extracted from the SIDER database (http://sideeffects.embl.de/) (last updated on 15 October 2015). In the GIPADD database, 95 out of 172 drugs were also listed in the SIDER database. The datasets associated with the remaining 77 drugs, which were not included in any training set, were used for external validation. The targets for the classification model were defined as follows. For a given ADR, datasets associated with ADR-inducing drugs were labelled as *class 1*, whereas those associated with drugs not known to induce ADRs were labelled as *class 0*. When a drug was labelled as *INDICATION* by SIDER for a specific ADR, the corresponding target was adjusted to *class 0* to prevent false-positive labels.

### Data balance and splitting

For each ADR, balanced subsets (*class 0* and *class 1*) of all datasets (termed balanced chunks) were prepared for nausea, vomiting, and diarrhoea prediction models but not for the constipation prediction model because its training set was already nearly balanced. These datasets were divided into training and validation datasets at a ratio of 8:2 through random sampling. The splitting was conducted at the dataset level, with each dataset representing raw data collected from a single independent electrophysiology experiment, which includes a 5-min baseline recording followed by a 7-min post-drug recording. Please refer to Supplementary Table 1 for the number of datasets used in each trained model for this study. The splitting process is independent of the types of tissues or concentrations tested; these parameters will be incorporated as embedding layers in the training model. A balanced chunk included all datasets from the minority class and some datasets drawn randomly from the majority class, ensuring no overlap in majority samples between any two balanced chunks for the same ADR. To determine whether the trained model can identify features correlated with the ADR, we trained the model in a setting in which the labels of the training datasets were shuffled prior to training (a negative correlation control). Subsequently, the performance of the model trained using shuffled labels was compared with that of models trained using correct labels (positive correlation).

### Training models

A simple convolutional neural network (CNN, *CNNClassifier*), FCN (*FCNClassifier)*^19^, and ICT (*IndividualInceptionClassifier*)^20^ adopted from *aeon-toolkit.org* (0.5.0 documentation) and a Python library for time-series classification models, were tested and compared. To include covariate information during model training, we introduced fixed embedding layers for both tissues and concentrations (t and c, respectively). The vector output from these embedding layers were added to the input before forward propagation. Each model was trained five times, resulting in five independent classifiers for each data chunk and ADR, using mini-batch stochastic gradient descent (with a batch size of 30) and the Adam optimiser for 200 epochs. All other parameters and settings of the trained model were adopted from *aeon-toolkit.org*. For each ADR and data chunk, we constructed an ensemble model from the five independent classifiers using a simple logistic regression model fitted with the *class 1* probabilities from each classifier as features and the target labels from the validation set as targets. The criterion is that a greater weight will be assigned to each independent classifier that demonstrates higher accuracy on the validation datasets. During inference, the individual classifiers received filtered recordings and the covariates as inputs, whereas the ensemble model received *class 1* probabilities from the multiple classifiers as inputs.

### Predictions

The prediction output from all models was a two-component vector with non-negative real values between 0 and 1, representing the probabilities of a dataset belonging to *class 0* or *class 1*. To minimise biological variance, the prediction probabilities of datasets representing the same drug were averaged or clustered by tissue type (t) and concentration (c) to evaluate model performance. A classification cutoff of 0.5 was applied, categorising results into *class 0* (< 0.5) and *class 1* (≥ 0.5) for model evaluation. Model performance was assessed using accuracy, precision for *class 1*, and area under the receiver operating characteristic curve (AUROC), with evaluations based on forward-shifted (490–690 s) and backward-shifted (510–710 s) post-drug matrices combined with the original baseline matrix (Fig. 2C). The time-shifted matrices constituted completely different input ‘interference’ matrices, diverging from the training matrix. The performances of different models were compared using Student’s tests, with a *p* value < 0.05 indicating statistical significance.

## Results and discussion

### Basic model performance (by dataset)

The average (by dataset) accuracy, precision (*class 1*), and AUROC for the validation datasets in the trained models (*n* = 5) are listed in Supplementary Table 1. The performance metrics based on the validation datasets provide limited insights into the model’s ability to generalise for datasets associated with unseen drugs because there is no control for biological variance.

### Model performance evaluation using time-shifted external validation datasets

Each dataset alone does not fully represent the drug’s performance, particularly in the context of applying AI to physiological questions, where biological variances can lead to significant variations in results. In our experiments, we intend to use replicates from different test sessions to dual with the problem in biological variances. We decided to use summary statistics of the by-dataset prediction results for classification. To do this, we introduced the concept of ‘by-drug prediction probabilities’, where the prediction probability of a drug is calculated as the average of the by-dataset prediction probabilities across all datasets corresponding to the drug. We also think that using only the 20% validation datasets to average by-drug predictions will lead to imbalanced data representations for each drug. Therefore, in our evaluation, we used time-shifted datasets for external validation. These time-shifted matrices were generated by adding a time-shifted post-drug matrix to the original baseline matrix (Fig. 2C), thus creating a distinct matrix for prediction. We evaluated the models using two time-shifted datasets named time-shifted-4969 and time-shifted-5171. The time-shifted-4969 dataset contained backward-shifted post-drug recordings, whereas the time-shifted-5171 dataset contained forward-shifted post-drug recordings.

Models trained with correctly labelled data (positive correlation) performed significantly better than those trained with shuffled labels (negative correlation) in all ICT classifiers, demonstrating higher accuracy, precision (*class 1*), and AUROC values (*p* < 0.05, unpaired Student’s t tests; *n* = 20, for nausea, vomiting, and diarrhoea prediction models, which consisted of two chunks, five classifiers, and two time-shifted prediction datasets; *n* = 10 for constipation prediction models, which consisted of one chunk, five classifiers, and two time-shifted prediction datasets; Fig. 1). This result indicates that the ICT classifiers successfully learned ADR-related features and applied them effectively to time-shifted validation datasets. However, the FCN model did not exhibit improved accuracy in prediction models for nausea, vomiting, or diarrhoea between negative and positive datasets (*p* < 0.05, *n* = 20, unpaired Student’s t tests). Similarly, the CNN model did not show improved precision (*class 1*) in prediction models for nausea or diarrhoea (*p* < 0.05, *n* = 20, unpaired Student’s t tests). This finding indicates that the current FCN and CNN models are not effective at classifying drugs based on the target ADRs.

Ensemble models were developed on the basis of the validation results of the five ICT classifiers. All ensemble models performed significantly better than the five individual classifiers, demonstrating higher accuracy (*p* < 0.0001), precision (*class 1*; *p* < 0.05), and AUROC values (*p* < 0.001; paired Student’s t test). These results were observed for models addressing nausea, vomiting, and diarrhoea (*n* = 10) and constipation (*n* = 5; Fig. 5A). Details regarding the performance of the ensemble models, the top three ICT models, and the best shuffled negative control model are listed in Table 2, and the receiver operating characteristic curves for these models are illustrated in Fig. 4. The prediction results of the best ensemble models are available in Supplementary Tables 2 to 5.

**Table 2:** Summary table showing the model performance of best three *InceptionTime* (ICT) models, best shuffled negative control model, and the ensemble model trained based on 5 ICT classifiers. The score of the model was calculated as [(Accuracy + Precision (*class 1*) + AUROC)/3]. The model with the highest score was ranked as the best three ICT models. The same models listed in this table were used for plotting AUROC curve in Fig. 3. *AUROC* area under the receiver operating characteristic curve.

ADR	Chunk	Model	Labelling	Prediction dataset	Tissue data used	Accuracy	Precision (class 1)	AUROC	Score
Nausea	0	Ict-tr5	Correct	4969	All tissues	0.809	0.864	0.862	0.845
	0	Ict-tr1	Correct	5171	All tissues	0.843	0.878	0.813	0.845
	0	Ict-tr5	Correct	5171	All tissues	0.817	0.857	0.860	0.845
	0	Ict-ensemble	Correct	5171	All tissues	0.870	0.882	0.835	0.862
	0	Ict-ensemble	Correct	4969	All tissues	0.861	0.880	0.829	0.857
	0	Ict	Shuffled	5171	All tissues	0.739	0.746	0.472	0.652
Vomiting	1	Ict-tr4	Correct	5171	All tissues	0.843	0.898	0.846	0.863
	1	Ict-tr5	Correct	4969	All tissues	0.852	0.866	0.857	0.858
	0	Ict-tr2	Correct	5171	All tissues	0.861	0.853	0.851	0.855
	1	Ict-ensemble	Correct	4969	All tissues	0.887	0.903	0.869	0.886
	1	Ict-ensemble	Correct	5171	All tissues	0.852	0.882	0.875	0.870
	1	Ict	Shuffled	5171	All tissues	0.765	0.765	0.370	0.633
Diarrhoea	0	Ict-tr3	Correct	5171	All tissues	0.809	0.956	0.895	0.887
	0	Ict-tr3	Correct	4969	All tissues	0.800	0.930	0.902	0.877
	1	Ict-tr2	Correct	4969	All tissues	0.696	0.945	0.885	0.842
	0	Ict-ensemble	Correct	4969	All tissues	0.852	0.986	0.937	0.925
	0	Ict-ensemble	Correct	5171	All tissues	0.843	0.985	0.915	0.915
	0	Ict	Shuffled	5171	All tissues	0.722	0.736	0.488	0.649
Constipation	0	Ict-tr3	Correct	5171	All tissues	0.835	0.917	0.935	0.895
	0	Ict-tr4	Correct	5171	All tissues	0.783	0.940	0.917	0.880
	0	Ict-tr4	Correct	4969	All tissues	0.739	0.953	0.907	0.867
	0	Ict-ensemble	Correct	5171	All tissues	0.913	0.893	0.958	0.921
	0	Ict-ensemble	Correct	4969	All tissues	0.896	0.890	0.957	0.914
	0	Ict	Shuffled	5171	All tissues	0.617	0.626	0.634	0.626

**Fig. 3:**
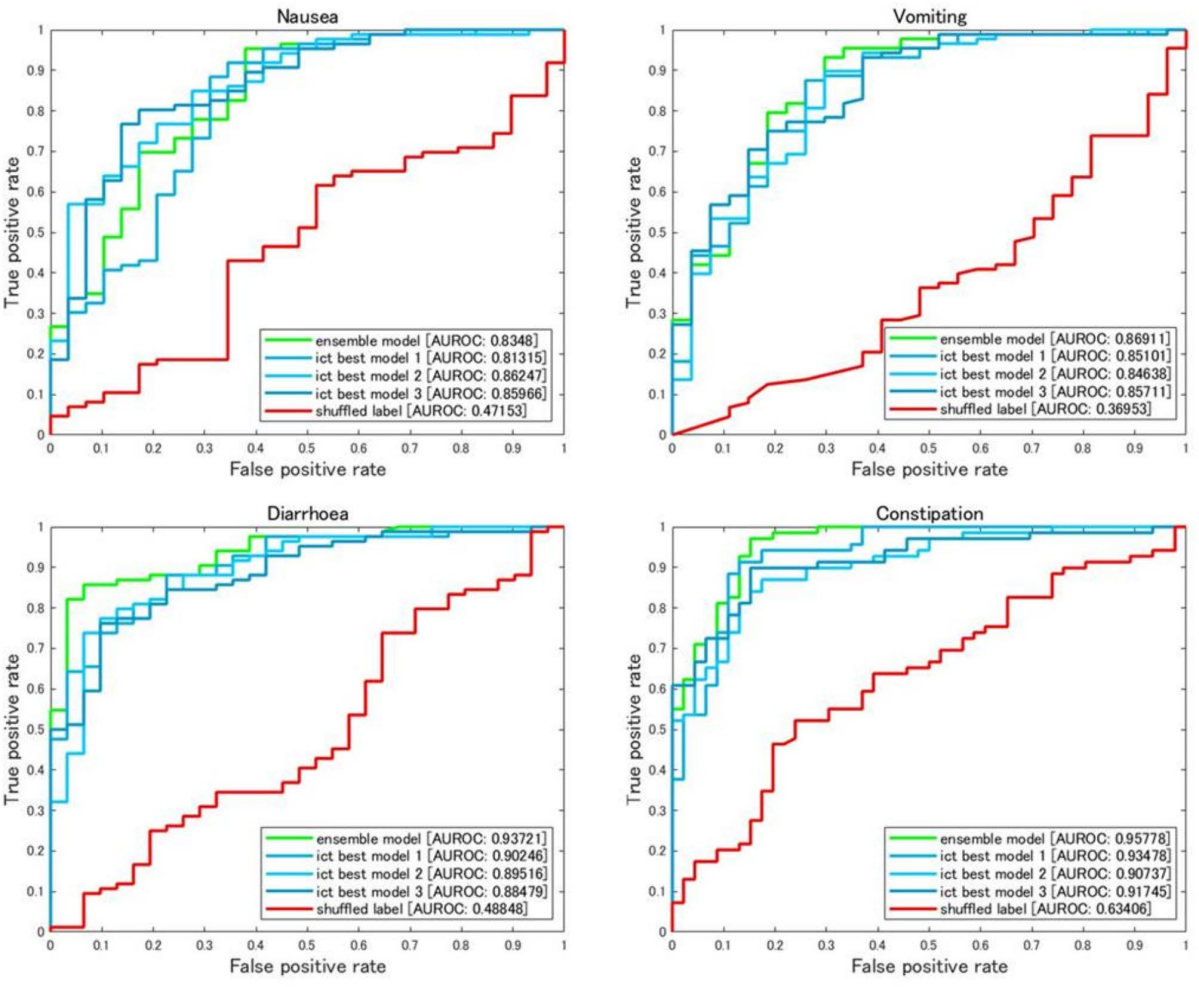
Receiver operating characteristic curves for the top three ICT models, the best ensemble model, and the best shuffled label negative control ICT model for nausea, vomiting, diarrhoea, and constipation. *ICT* InceptionTime classifier.

**Fig. 4:**
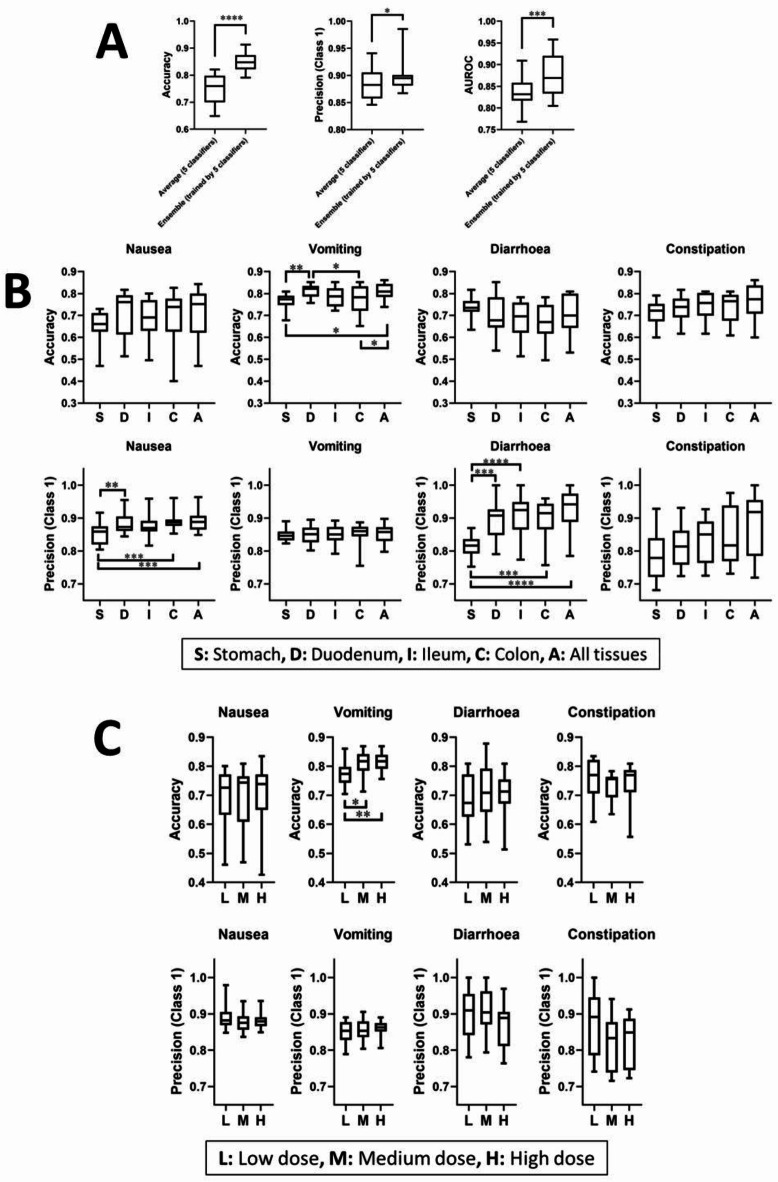
(**A**) Performance of ensemble models. Data represent the mean ± standard deviation. Significant differences in performance between the average of five InceptionTime (ICT) classifiers and the ensemble model trained based on the five ICT classifiers are indicated as *p < 0.05, **p < 0.01, ***p < 0.001, and ****p < 0.001 (paired Student’s t tests). (**B**) Model performance differentiated by gut tissue types. Data represent the mean ± standard deviation, and significant differences are indicated as *p < 0.05, **p < 0.01, ***p < 0.001, and ****p < 0.001 (Tukey’s multiple comparisons test). (**C**) Model performance differentiated by the tested drug concentration (low, medium, and high doses). Data represent the mean ± standard deviation, and significant differences are indicated as *p < 0.05, **p < 0.01, ***p < 0.001, and ****p < 0.001 (Tukey’s multiple comparisons test), where *n* = 20 for nausea, vomiting, and diarrhoea classifiers consist of two chunks, five classifiers, and two time-shifted prediction datasets and *n* = 10 for constipation classifiers consist of one chunk, five classifiers, and two time-shifted prediction datasets.

### Tissue- and concentration-dependent effects

**Fig. 5:**
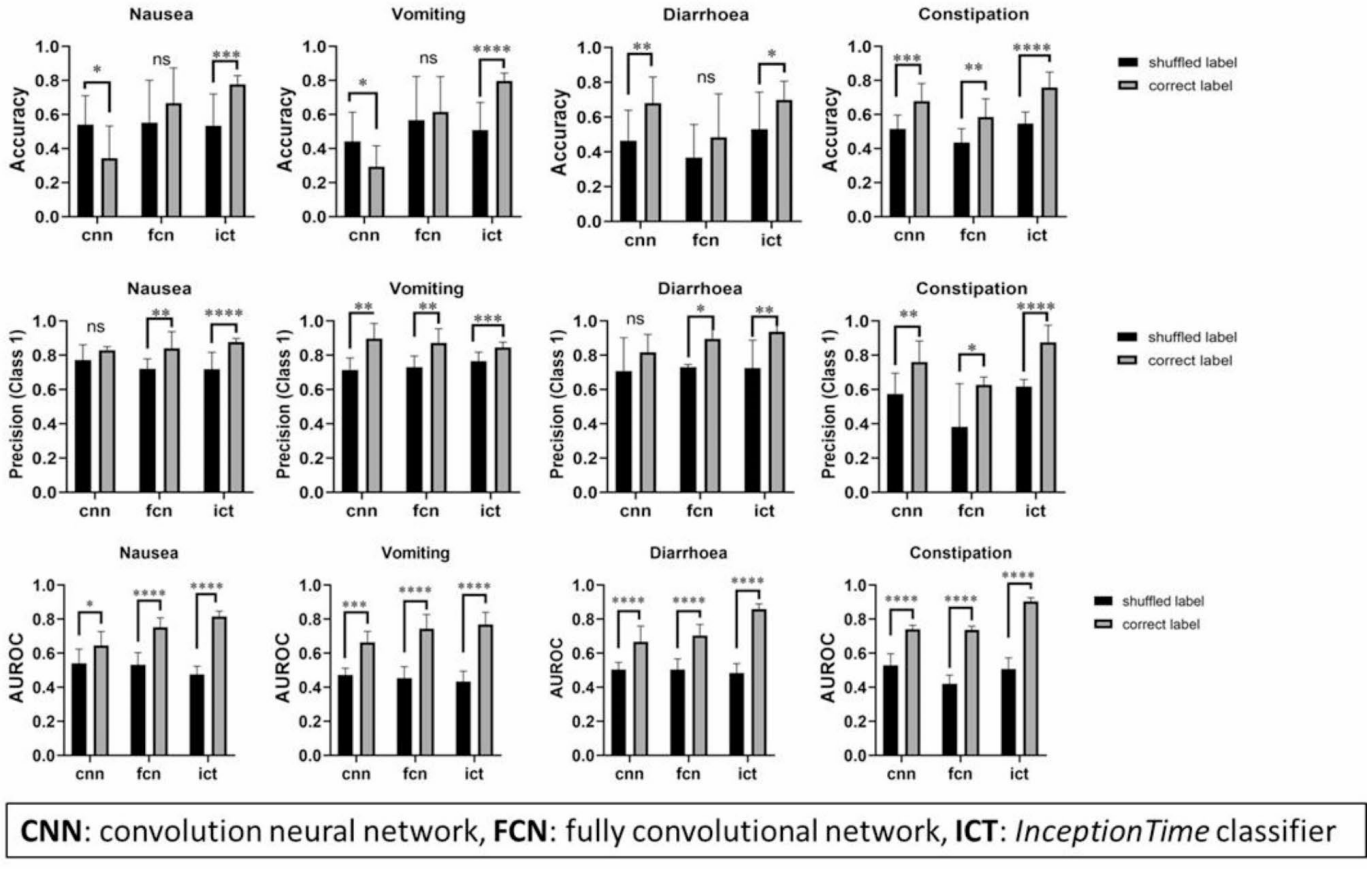
Performance of models trained with correct label (positive correlation) and shuffled label (negative correlation) datasets, using CNN, FCN, and ICT deep-learning classifiers. Data represent the mean ± standard deviation, and significant differences in model performance between positive (correct) and negative (shuffled) labels are indicated as *p < 0.05, **p < 0.01, ***p < 0.001, and ****p < 0.001 (unpaired Student’s t tests), where *n* = 20 for nausea, vomiting, and diarrhoea classifiers consist of two chunks, five classifiers, and two time-shifted prediction datasets and *n* = 10 for constipation classifiers consist of one chunk, five classifiers, and two time-shifted prediction datasets. *CNN* convolutional neural network, *FCN* fully convolutional network, *ICT* InceptionTime classifier.

In “Model performance evaluation using time-shifted external validation datasets”, we described the determination of the performance indices by averaging all datasets corresponding to the same drugs. In this section, we described the exploration of whether prediction accuracy improves when we average across all datasets collected from specific gut tissue segments and an examination of the impact of the test drug concentration on prediction performance. For simplicity, we evaluated only accuracy and precision (*class 1*). In the vomiting prediction models, the accuracy was significantly higher for datasets derived from the duodenum (0.815 ± 0.031, *n* = 20) than those derived from the stomach (0.769 ± 0.032, *p* < 0.01, *n* = 20) and colon (0.771 ± 0.064, *p* < 0.05, *n* = 20). Combining data from all tissue types resulted in an accuracy of 0.809 ± 0.037 (*n* = 20), which outperformed the accuracy for data from the stomach (*p* < 0.05) and colon (*p* < 0.05; Fig. 5B). This finding indicates that the duodenal data appeared to have better predictive power for vomiting-inducing properties than the stomach or colon data. For nausea prediction models, the precision (*class 1*) was significantly lower in the stomach (0.852 ± 0.032, *n* = 20) than in the duodenum (0.883 ± 0.028, *p* < 0.01, *n* = 20), colon (0.890 ± 0.023, *p* < 0.001, *n* = 20), and all tissues combined (0.891 ± 0.030, *p* < 0.001, *n* = 20). Similarly, for diarrhoea prediction models, precision (*class 1*) was significantly lower in the stomach (0.813 ± 0.034, *n* = 20) than in the duodenum (0.896 ± 0.059, *p* < 0.001, *n* = 20), ileum (0.904 ± 0.069, *p* < 0.0001, *n* = 20), colon (0.894 ± 0.066, *p* < 0.001, *n* = 20), and all tissues combined (0.920 ± 0.070, *p* < 0.0001, *n* = 20; Fig. 3). These findings indicate that the drug-induced changes in pacemaker activities in the stomach are less predictive of nausea and diarrhoea compared with those in intestinal segments.

In terms of dose-dependent effects, only the vomiting model exhibited a significantly lower accuracy for low-dose datasets (0.775 ± 0.040, *n* = 20) than medium-dose datasets (0.807 ± 0.045, *p* < 0.05, *n* = 20) and high-dose datasets (0.813 ± 0.033, *p* < 0.05, *n* = 20; Fig. 5C). This finding indicates that prediction accuracy for identifying a drug’s vomiting-inducing potential is sensitive to the tested drug dose, given that all drugs were tested at their known effective doses (rounded to the nearest exponent, e.g. 10^− 3^ M and 10^− 6^ M) for the activation or inhibition of their targeted receptors and at doses 10-fold lower and higher than the effective level to enrich the database with a detailed drug profile related to GIPA. More information about the used doses of the tested drugs can be found at http://www.gutrhythm.com/public_database. Higher- and lower-concentration tests were only conducted on intestinal segments, not the stomach, because of the limited amount of stomach tissue available per animal. Thus, the total number of datasets extracted from the stomach was approximately three times smaller than the number of intestinal datasets for the aforementioned analyses.

### Prediction results for selected drugs (not used in training)

Seventy-seven drugs that did not have corresponding profiles in the SIDER database were excluded from the training process to be used as part of the external drug validation. We evaluated a specific set of drugs that were not included in the training phase using the two most effective ensemble models for each ADR. The results of these evaluations are summarized in Table 3. The prediction results of all tested drugs can be found in Supplementary Table 6.

**Table 3:** Prediction results for selected drugs (not used in training). Prediction values are the probability of the drug to be *class 1* averaging all tissues and all drug concentration predicted using two most effective ensemble models for each ADR. Prediction values above 0.7 are bolded.

Drug name	Treatment	Nausea	Vomiting	Diarrhoea	Constipation
Rolipram	Antidepressants—PDE4 inhibitors Discontinued due to gut side effects	**0.86**	**0.82**	**0.89**	0.31
Lixisenatide	Diabetes—GLP1 agonist Discontinued due to gut side effects	**0.77**	**0.75**	0.52	0.43
Exendin-4	Diabetes—GLP1 agonist	**0.73**	**0.74**	0.58	0.47
Dulaglutide	Diabetes—GLP1 agonist	**0.72**	**0.84**	0.59	0.54
Vitamin C	Common food supplement	0.63	0.59	0.35	0.50
Danuglipron	Pfizer—diabetes—GLP1 agonist Recently halt twice-daily dose due to nausea and vomiting	0.57	0.59	0.28	0.47
Krebs	negative control	0.47	0.58	0.50	0.50
Acyl-ghrelin	Appetite enhancing hormone	0.37	0.37	0.49	0.69

We selected four GLP1 agonists for our evaluation: dulaglutide (Trulicity, developed by *Eli Lilly*), lixisenatide (Lyxumia, developed by *Sanofi*), exendin-4 (also known as exenatide, developed by multiple companies), and danuglipron (developed by Pfizer). Dulaglutide induces nausea, vomiting, and diarrhoea in at least 5% of patients^21^, and our model predicted values of 0.72, 0.85, and 0.64, respectively, for these ADRs. We currently consider a prediction value greater than 0.7 for *class 1* as indicative of risk, although a more precise risk threshold may be established in the future when more external drug validation data are available. Lixisenatide, which was discontinued due to ADRs such as nausea (> 20%) and vomiting (> 9%)^22^, showed prediction values of 0.80 and 0.78 for nausea and vomiting, respectively. Exendin-4 was reported to cause mild to moderate nausea, vomiting, and diarrhoea in clinical trials^23^, and it had prediction values of 0.76, 0.76, and 0.59, respectively, for these ADRs in our study. Pfizer recently decided not to advance a twice-daily dose of danuglipron due to high discontinuation rates resulting from nausea and vomiting. Studies testing a once-daily dose are ongoing. Our prediction values for this drug were 0.56 and 0.60 for nausea and vomiting, respectively. These values are not considerably higher than those of other tested GLP1 agonists. More data will be required to accurately assess its long-term clinical performance.

Rolipram, a PDE4 inhibitor previously discontinued due to GI side effects, including nausea and diarrhoea^24^, showed prediction values of 0.89 and 0.90, respectively, in our model. Notably, the prediction value for vomiting for rolipram also reached 0.85.

As negative controls, we included vitamin C (l-ascorbic acid, a common food supplement), the appetite-enhancing hormone acyl ghrelin, and Krebs’ solution (a medium used in experiments). None of these controls exhibited a prediction value greater than 0.7 for nausea, vomiting, diarrhoea, or constipation.

### Advantages over current preclinical models to predict nausea and vomiting

Predicting nausea and vomiting currently requires testing in preclinical live animal models to observe vomiting behaviour^6,7^. Although the proposed drug screening prediction assay necessitates freshly isolated animal tissues, the electrophysiology experiment and predictions can be completed within a day. Moreover, in addition to nausea and vomiting, the proposed assay can be used to predict diarrhoea and constipation and even ADRs affecting areas outside the GI tract.

### Limitations

The ADR annotations in the SIDER database are incomplete and incorrect for some drugs^25^, necessitating an update to achieve more accurate labels before model training. Another limitation is that the prediction results of the current models were based on the similarity of the profiles of novel drugs to those of known ADR-inducing drugs. This comparison provides an estimate of the likelihood that the new drug could cause an ADR, instead of an exact probability of such ADRs occurring in clinical trials. Finally, we used time-shifted datasets for external validation. Although these time-shifted datasets are entirely different from the training input, both numerically and at waveforms level, the potential bias introduced by this validation method remains unknown.

## Conclusions and future studies

We successfully used a state-of-the-art time-series deep-learning classification model, the ICT, on the GIPADD to develop predictive models for drug-induced nausea, vomiting, diarrhoea, and constipation. However, there is still considerable scope for enhancing these models, including optimising hyperparameters, refining training layers, and exploring various input transformations.

In the future, we will (i) test different transformations of the input matrix to identify various drug-induced features, such as subtracting post-drug recordings from baseline recordings; (ii) represent our data in a graph format to apply models suited for spatiotemporal data (e.g. spatiotemporal graph CNNs)^26^, (iii) use other signal transformations, including the power spectrum, wavelet transform, and Hilbert transform; and (iv) integrate all of the aforementioned features to train a large-scale ensemble model.

We also plan to expand the GIPADD by incorporating data from other organs, such as the uterus, pancreas, bladder, and gallbladder, in addition to gut tissues. We have also trained models to predict other ADRs outside the GI tract. As the GIPADD continues to grow with more datasets and a broader array of tested drugs, it may not only predict outcomes other than ADRs. With the availability of more high-quality data, the GIPADD may be able to predict drug activation or inhibition effects, which may be correlated to therapeutic outcomes. The expansion of the GIPADD opens up numerous possibilities for future research in drug discovery and safety evaluation.

In this manuscript, we present predictions for only four types of ADRs as a proof of concept. We are actively exploring the potential to predict more than 100 types of ADRs. For initial exploration, we considered a minimum “by-drug” accuracy of 70% acceptable during the early stages of experimentation. Once we confirm the predictive power for specific types of ADRs using the GIPADD database, we expect to increase the minimum “by-drug” accuracy to at least 80%.

Finally, the interpretation of ‘risk’ can vary significantly depending on the context, especially concerning ADRs. For instance, cardiac risks should be approached with much greater caution than gastrointestinal risks. Different experts may interpret threshold risk values in various ways, making it advantageous to maintain flexibility in the interpretation of AI-predictive results at this stage. We expect these risk values would provide pharmaceutical companies with useful information to inform their decisions, such as incorporating anti-GI side effect considerations into clinical trial designs or selecting drug candidates that may have lower efficacy but more favourable side effect profiles during the drug development process. Further experimentation and research on a range of drugs will be essential to confirm and validate the risk values predicted by our models.

## Electronic supplementary material

Below is the link to the electronic supplementary material.

Supplementary Material 1

Supplementary Material 2

Supplementary Material 3

Supplementary Material 4

Supplementary Material 5

Supplementary Material 6

## Data Availability

The analysed data collected are available as open data via the Gut Rhythm R&D (Hong Kong) Limited online data repository: https://www.gutrhythm.com/public_database.The raw data that support the findings of this study are available from Julia Yuen Hang Liu but restrictions apply to the availability of these data, which were used under license from the Gut Rhythm R&D (Hong Kong) Limited for the current study, and so are not publicly available. Data are, however, available from Julia Yuen Hang Liu upon reasonable request and with permission from the Gut Rhythm R&D (Hong Kong) Limited.

## Abbreviations

GIPADD: Gastro-intestinal pacemaker activity drug database
SIDER: Side effect resource (http://sideeffects.embl.de/)
ML: Machine learning
ADRs: Adverse drug reactions
FFT: Fast Fourier transform
GI: Gastrointestinal
ICCs: Interstitial cells of Cajal
AI: Artificial intelligence
sf: Sampling frequency
CNN: Convolutional neural network
FCN: Fully convolutional network
ICT: InceptionTime classifier
