# Supplementary material for: An application of deep learning model InceptionTime to predict nausea, vomiting, diarrhoea, and constipation using the gastro-intestinal pacemaker activity drug database (GIPADD)
Source: Sci Rep. 2025 Apr 16;15:13105. doi: 10.1038/s41598-025-95961-4 (PMC12003867; doi:10.1038/s41598-025-95961-4)
Supplement: Supplementary file 6 — Supplementary Material 6 [file 41598_2025_95961_MOESM6_ESM.docx]

| ADR | Chunk | Training datasets | | Validation datasets | | Model | Accuracy | Precision (class 1) | AUROC |
| --- | --- | --- | --- | --- | --- | --- | --- | --- | --- |
|  |  | Class 0 | Class 1 | Class 0 | Class 1 |  |  |  |  |
| Nausea | 0 | 1451 | 1444 | 358 | 365 | CNN | 0.607 ± 0.043 | 0.619 ± 0.053 | 0.639 ± 0.064 |
|  |  |  |  |  |  | FCN | 0.610 ± 0.054 | 0.630 ± 0.065 | 0.649 ± 0.067 |
|  |  |  |  |  |  | ICT | 0.623 ± 0.028 | 0.630 ± 0.030 | 0.666 ± 0.043 |
| Nausea | 1 | 1452 | 1443 | 357 | 366 | CNN | 0.600 ± 0.042 | 0.610 ± 0.044 | 0.639 ± 0.062 |
|  |  |  |  |  |  | FCN | 0.611 ± 0.055 | 0.622 ± 0.063 | 0.644 ± 0.080 |
|  |  |  |  |  |  | ICT | 0.622 ± 0.043 | 0.639 ± 0.056 | 0.667 ± 0.061 |
| Vomiting | 0 | 1326 | 1374 | 361 | 313 | CNN | 0.641 ± 0.073 | 0.594 ± 0.063 | 0.694 ± 0.104 |
|  |  |  |  |  |  | FCN | 0.647 ± 0.079 | 0.616 ± 0.083 | 0.696 ± 0.108 |
|  |  |  |  |  |  | ICT | 0.680 ± 0.066 | 0.646 ± 0.071 | 0.724 ± 0.083 |
| Vomiting | 1 | 1347 | 1353 | 340 | 334 | CNN | 0.567 ± 0.016 | 0.562 ± 0.020 | 0.591 ±0.018 |
|  |  |  |  |  |  | FCN | 0.675 ± 0.010 | 0.678 ± 0.007 | 0.723 ± 0.015 |
|  |  |  |  |  |  | ICT | 0.683 ± 0.013 | 0.681 ± 0.016 | 0.742 ± 0.009 |
| Diarrhoea | 0 | 1396 | 1385 | 342 | 353 | CNN | 0.583 ± 0.061 | 0.601 ± 0.067 | 0.622 ± 0.078 |
|  |  |  |  |  |  | FCN | 0.586 ± 0.056 | 0.593 ± 0.055 | 0.617 ± 0.080 |
|  |  |  |  |  |  | ICT | 0.615 ± 0.058 | 0.619 ± 0.057 | 0.651 ± 0.073 |
| Diarrhoea | 1 | 1391 | 1390 | 347 | 348 | CNN | 0.584 ± 0.050 | 0.595 ± 0.056 | 0.610 ± 0.056 |
|  |  |  |  |  |  | FCN | 0.555 ± 0.065 | 0.552 ± 0.063 | 0.581 ± 0.084 |
|  |  |  |  |  |  | ICT | 0.587 ± 0.052 | 0.585 ± 0.051 | 0.634 ± 0.065 |
| Constipation | 0 | 2284 | 2286 | 572 | 570 | CNN | 0.596 ± 0.076 | 0.599 ± 0.084 | 0.633 ± 0.102 |
|  |  |  |  |  |  | FCN | 0.601 ± 0.068 | 0.600 ± 0.070 | 0.634 ± 0.092 |
|  |  |  |  |  |  | ICT | 0.613 ± 0.061 | 0.619 ± 0.065 | 0.655 ± 0.089 |

**Supplementary-table-1. Table showing the model performance (by datasets)**. Data represent the mean ± standard deviation of five independent trained classifiers using the same training datasets and predicted using the internal validation datasets. Class 0 represents datasets tested with drugs that are not known to induce the ADR, and class 1 represents datasets tested with drugs that are known to induce the ADR clinically. ADR: adverse drug reactions; CNN: convolutional neural network; FCN: fully convolutional network; ICT: *InceptionTime* classifier.

**Supplementary-table-2.** An excel table named “Supplementary table 2 ADR_Nausea_chunk_0_ict_5171_ensemble.csv”, showing the “by-dataset” prediction results of the ict-ensemble model for predicting nausea using time-shifted-5171 as the prediction datasets.

**Supplementary-table-3.** An excel table named “Supplementary table 3 ADR_Vomiting_chunk_1_ict_4969_ensemble.csv”, showing the “by-dataset” prediction results of the ict-ensemble model for predicting vomiting using time-shifted-4969 as the prediction datasets.

**Supplementary-table-4.** An excel table named “Supplementary table 4 ADR_Diarrhoea_chunk_0_ict_4969_emsemble.csv”, showing the “by-dataset” prediction results of the ict-ensemble model for predicting diarrhoea using time-shifted-4969 as the prediction datasets.

**Supplementary-table-5.** An excel table named “Supplementary table 5 ADR_Constipation_chunk_0_ict_5171_emsemble.csv”, showing the “by-dataset” prediction results of the ict-ensemble model for predicting constipation using time-shifted-5171 as the prediction datasets.

**Supplementary-table-6.** An excel table named “Supplementary table 6 by-drug prediction results for all tested drugs_v4.csv”, showing the prediction results for all tested drugs using the selected ensemble models. The prediction results presented are the averages obtained from the time-shifted-4191 and time-shifted-5171 datasets.
